# Incorporating Mental Health Research into Disaster Risk Reduction: An Online Training Module for the Hazards and Disaster Workforce

**DOI:** 10.3390/ijerph18031244

**Published:** 2021-01-30

**Authors:** Candace M. Evans, Rachel M. Adams, Lori Peek

**Affiliations:** 1 Natural Hazards Center and CONVERGE, Department of Sociology, University of Colorado Boulder, Boulder, CO 80309, USA; Lori.Peek@colorado.edu; 2 Natural Hazards Center and CONVERGE, University of Colorado Boulder, Boulder, CO 80309, USA; Rachel.Adams-1@colorado.edu

**Keywords:** disasters, disaster risk reduction, disaster mental health, psychosocial well-being, risk factors, training, education, workforce development

## Abstract

There is an expansive and growing body of literature that examines the mental health consequences of disasters and large-scale emergencies. There is a need, however, for more explicit incorporation of mental health research into disaster risk reduction practices. Training and education programs can serve as a bridge to connect academic mental health research and the work of disaster risk reduction practitioners. This article describes the development and evaluation of one such intervention, the CONVERGE Disaster Mental Health Training Module, which provides users from diverse academic and professional backgrounds with foundational knowledge on disaster mental health risk factors, mental health outcomes, and psychosocial well-being research. Moreover, the module helps bridge the gap between research and practice by describing methods used to study disaster mental health, showcasing examples of evidence-based programs and tools, and providing recommendations for future research. Since its initial release on 8 October 2019, 317 trainees from 12 countries have completed the Disaster Mental Health Training Module. All trainees completed a pre- and post-training questionnaire regarding their disaster mental health knowledge, skills, and attitudes. Wilcoxon Signed Rank tests demonstrated a significant increase in all three measures after completion of the training module. Students, emerging researchers or practitioners, and trainees with a high school/GED education level experienced the greatest benefit from the module, with Kruskal–Wallis results indicating significant differences in changes in knowledge and skills across the groups. This evaluation research highlights the effectiveness of the Disaster Mental Health Training Module in increasing knowledge, skills, and attitudes among trainees. This article concludes with a discussion of how this training can support workforce development and ultimately contribute to broader disaster risk reduction efforts.

## 1. Introduction

Disasters disrupt entire communities and cause widespread destruction, injury, and displacement. Such traumatic experiences can overwhelm regular coping capacity and available resources, contributing to adverse mental health outcomes among adults as well as children [1,2,3,4,5]. Decades of research on the mental health aspects of disaster indicate that the specific nature of acute, as well as chronic, outcomes is shaped by a number of individual- and societal-level characteristics and conditions that exist before, during, and after disaster [5]. For example, people experiencing poverty, residents of developing countries, children, and middle-aged adults are all at increased risk for adverse mental health outcomes in disaster [3,4,6,7,8]. Developing a skilled workforce that is trained to understand and respond to the root causes and complex consequences of disasters is therefore imperative for the development and implementation of effective policies and programs. 

In light of the differential risk experienced by people in diverse cultural and geographic contexts, global disaster policy frameworks, such as the United Nations’ Sendai Framework for Disaster Risk Reduction [9] and the World Health Organization’s Health Emergency and Disaster Risk Management Framework [10], have underscored the need for more explicit incorporation of mental health and psychosocial well-being research into disaster risk reduction (DRR) activities. DRR, as both a practice and a concept, emphasizes the reduction in the physical, social, and economic consequences of disaster through prevention [9]. Rather than solely focusing on response and recovery efforts, DRR promotes individual and collective actions that foster preparedness and mitigation activities *before* a disaster occurs. 

While considerable shifts towards DRR have occurred within the disaster and emergency management fields over recent decades [11], comparative efforts towards risk reduction within the mental health and psychosocial services fields are still in their infancy [12]. Indeed, in their recent review and mapping of the integration of mental health and psychosocial support with DRR, Gray and colleagues observe that scholars and practitioners within these fields typically focus on the response and recovery phases of a disaster and are significantly less involved in prevention and risk reduction efforts [12]. Moreover, there is a widespread lack of empirically based guidance for mental health service professionals to engage in disaster risk reduction practices, which further complicates efforts to heed global calls for more pronounced attention to mental health and psychosocial well-being in DRR efforts [13].

### Educating and Training the Disaster Risk Reduction Workforce

As disasters have grown in frequency and magnitude, so too has the demand for the knowledge and expertise of the researchers and practitioners working in this space [14,15]. Investing in the education of current and future generations of professionals prepared to integrate disaster mental health knowledge with disaster risk reduction strategies represents an opportunity for fostering convergence across organizational and disciplinary boundaries [15]. Moreover, training and education programs that focus on developing methodological, theoretical, and applied skills can help to ensure that the hazards and disaster workforce is prepared for increasingly complex 21st-century environmental challenges [14,16]. 

Such education and training programs must be holistic and designed in a way that recognizes the growing disciplinary, professional, and demographic diversity of the current and future workforce [17,18,19,20] while also addressing the continued underrepresentation of women, racial and ethnic minorities, and people from low-income communities and countries [21,22,23]. Training programs that are sensitive to these demographic shifts and workforce dynamics can help establish bridges between academic research, mental health service provision, and emergency management while cultivating a diverse and strong DRR sector [12,22]. Such programs can also equip members of the hazards and disaster workforce with the knowledge and skills to conduct extreme events research that is methodologically rigorous and ethically grounded, as well as attentive to the unique social, political, and cultural contexts in which disasters and hazard events occur [15,24]. This includes, among other things, a more comprehensive understanding of and training in the importance of integrating disaster mental health and risk reduction practices.

There is a clear and pressing need for additional training programs that bridge the divide between disaster mental health research and DRR activities. At present, however, available disaster mental health trainings often do not explicitly reference empirical evidence from past disaster studies nor include procedures and tools used for psychological screening and assessment [25,26]. Furthermore, even evidence-informed resources, such as psychological first aid [27] and the online disaster mental health trainings offered by the American Red Cross [28], focus narrowly on the skills needed for the immediate response to a sudden, acute-onset event. These initiatives do not consider the longer-term time horizons associated with hazard mitigation and disaster recovery, which are often of concern to DRR professionals. This represents an important gap, as it is essential that the hazards and disaster workforce has a strong background regarding the risk factors that shape disaster mental health outcomes across the disaster lifecycle, as well as access to actionable guidance on how research can be translated into practice.

In response to this identified knowledge gap and the need for more comprehensive training and education for current and future generations of researchers and practitioners, the CONVERGE initiative at the Natural Hazards Center at the University of Colorado Boulder partnered with the Centers for Disease Control and Prevention (CDC) to develop the CONVERGE Disaster Mental Health Training Module. This free online training module provides researchers and other professionals with an overview of mental health outcomes associated with disasters, such as post-traumatic stress disorder and depression, as well as specific risk factors that render certain populations more vulnerable to these outcomes. It describes methods used to study disaster mental health, showcases examples of evidence-based programs and tools, and provides recommendations for future research inquiries. In doing so, the module provides actionable guidance for researchers and professionals to engage in DRR practices that anticipate and reduce the mental health impacts of disasters and, ultimately, increase the resilience of individuals and communities. 

This article describes the creation and evaluation of the Disaster Mental Health Training Module. More specifically, we outline the unique ways that this training supports the translation of available research on disaster mental health into DRR practice. The evaluation then examines whether this novel training can effectively reach a diverse next generation of hazards and disaster workforce and increase relevant knowledge, skills, and attitudes among this population.

## 2. Materials and Methods 

The National Science Foundation-supported CONVERGE initiative seeks to advance ethical, coordinated, and scientifically rigorous convergence research by providing resources and connecting extreme events researchers across disciplines [15]. One of the major tasks of CONVERGE is to accelerate the training of a diverse next generation of hazards and disaster researchers and practitioners by developing and disseminating a variety of educational resources. These resources include a series of free, online training modules that are designed for students and others new to the field who hope to quickly background themselves on key information on a variety of topics [24]. In this section, we discuss the materials and methods used to develop and evaluate the Disaster Mental Health Training Module. This module is part of a larger series of trainings that expand upon other relevant research topics.

### 2.1. Literature Synthesis 

To develop a research-based training module on disaster mental health, two members of the CONVERGE research team (Evans and Adams) identified and reviewed highly cited literature that focused on mental health outcomes associated with disasters. Our goal was to identify both theoretical and empirical literature that offered a thorough overview of risk factors that contribute to poor disaster mental health outcomes. In addition, we sought research focused on resiliency, post-traumatic growth, and evidence-based interventions. As such, we searched for studies that used a variety of theoretical lenses and methodological approaches and focused on different mental health outcomes and populations exposed to disaster.

We used the search terms “mental health” AND “disaster” to find literature in both Google Scholar and Web of Science. We then downloaded the results from these searches into Google spreadsheets and organized the publications into the following categories: (1) full article citation; (2) number of citations; (3) year of publication; (4) search engine/database; and (5) abstract. Next, as part of our data cleaning, we completed a review of the abstracts to eliminate duplicates, non-English language publications, and publications that did not focus on mental health outcomes associated with disaster events. 

We then organized the remaining citations according to document type (empirical research article; theoretical/review article; book/book chapter; or report) and downloaded the top 25 most cited publications of each document type into separate spreadsheets, where we added additional columns to inventory the publications based on their empirical content, methodological approach, and type of disaster studied. Once the annotated bibliography spreadsheets were complete, our team reviewed the reference lists of key readings to identify additional relevant literature that did not emerge in the original searches. This helped to ensure comprehensive coverage of the disaster mental health literature. As a result of this multi-step process, we selected 96 publications to inform the development of the training module.

### 2.2. Writing and Development

After finalizing the literature database, the same two members of the team (Evans and Adams) read all the relevant publications and worked to draft the sections of the module. Completed drafts were regularly presented to other members of our core team from the Natural Hazards Center and the CDC for feedback, suggestions, and edits, which were then incorporated into subsequent drafts. It took approximately six months to develop a full draft of the module. Once the draft was complete, graduate students and research associates at the Natural Hazards Center reviewed the written content and provided extensive feedback using a standardized review form. After the recommended revisions were made, disaster mental health subject matter experts at the CDC reviewed the content to ensure its face validity.

Next, we worked with a web developer with expertise in online learning technologies to transfer the content to an interactive online learning management system, LearnDash. LearnDash is a WordPress plugin created by leading learning industry professionals that provides practical and experience-driven guidance for individuals and organizations developing online courses. This allowed us to design the module based on best practices for e-learning and also permits our team to periodically make revisions and updates to the module, as new articles and books are published.

Once the online module was completely formatted in LearnDash, we asked members from the Natural Hazards Center’s research team to test the module. Each user tested the module on different devices and using different browsers to ensure its online functionality and operability before its release via the CONVERGE website.

### 2.3. Module Overview and Structure

The Disaster Mental Health Training Module summarizes decades of research on mental health in disasters while also offering practical recommendations for advancing research and practice on the topic. Specific methodological approaches and evidence-based tools are presented throughout the module to help guide the conduct and translation of rigorous disaster mental health research. The module interface features a progress bar and other navigational tools to ensure that learners know where they are in the module and can easily navigate through its various sections. To help ensure that trainees remain engaged as they move through the module, knowledge checks consisting of two true/false or multiple choice questions are included at the end of each major section. At the end of the module, trainees complete a final 10-question quiz. If they get at least 8 out of 10 questions correct on the assessment quiz, they receive a signed certificate indicating that they completed the CONVERGE module. The module takes approximately 30 to 60 min to complete and all content presented meets the accessibility standards required by the University of Colorado Boulder. 

The module is organized into four main lessons: (1) Background; (2) Methodological Approaches; (3) Knowledge to Action; and (4) Future Directions. Each of these lessons consists of different topics, which present interactive information that aims to achieve a specific set of learning objectives. For instance, by the end of the module it is expected that trainees are able to describe specific mental health outcomes that may emerge in the aftermath of a disaster or emergency; understand the conditions and risk factors that exist before, during, and after disaster that can influence mental health outcomes; and identify potentially vulnerable populations that may be at greater risk for poor mental health outcomes in the aftermath of a disaster. 

The first lesson, Background, introduces participants to the module by defining mental health within the context of various hazard types including natural hazards, technological disasters, and terrorism [29]. It then proceeds to summarize the risk factors and conditions that shape disaster mental health outcomes over time and across the disaster lifecycle [3]. Call-out boxes are also included throughout this lesson to highlight specific population groups that have been identified in the literature as especially vulnerable to poor disaster mental health outcomes, such as individuals with pre-existing mental health conditions, children, and low-income populations [3,4,5,30]. 

The next lesson, Methodological Approaches, describes common methodological approaches used by researchers to study mental health outcomes in a disaster context, including diagnostic screening tools [31,32], longitudinal survey research [33,34], qualitative methods [35,36], and mixed methods approaches [37]. Examples of past studies employing these different methods are emphasized as case examples. The lesson ends with a discussion of specific ethical considerations for studying disaster mental health to highlight the importance of ethical research practices.

The third lesson, Knowledge to Action, bridges research and practice by highlighting advances in evidence-based action and demonstrating how disaster mental health research can inform public health, emergency management, and DRR practice and policy. Evidence-based trainings, tools, and policies are presented in call-out boxes and as illustrative examples in this particular lesson. For instance, the module describes how research on the vulnerability of children to negative disaster mental health outcomes helped to inform the development of a number of CDC resources, such as the Helping Children Cope During and After a Disaster Factsheet [38]. It also describes how evidence-based interventions, such as Cognitive-Behavioral Therapy [5,39], can be incorporated into response and recovery efforts to address disaster mental health needs. At the end of the lesson, a logic model is used to summarize the connections between research, practice, and population outcomes.

The last lesson, Future Directions, provides 10 recommendations to help researchers address enduring challenges in the field and guide the development of new disaster mental health research questions and directions. The recommendations are drawn from widely cited research articles and reviews of disaster mental health scholarship [3,5,13], and center on the topics of (1) family- and community-level focus; (2) gene and environment interaction; (3) longitudinal assessments; (4) researcher-practitioner collaborations; (5) evidence-based interventions; (6) broader mental health outcomes focus; (7) participatory action research; (8) interdisciplinary collaborations; (9) intersectionality; and (10) mental health surveillance. 

In addition to the training content, the module also contains an additional resources tab where users can access a list of resources related to disaster mental health, including links to datasets, screening and diagnostic tools, key readings, and web resources. These supplementary materials are meant to support additional learning of the many and varied mental and psychosocial consequences that children and adults may experience in disaster. 

### 2.4. Access and Dissemination

After the Disaster Mental Health Training Module was tested and finalized, it was then published online via the CONVERGE website (https://converge.colorado.edu/resources/training-modules). Doing so allowed us to provide the module as a free resource to interested parties, though prior registration to the site is required in order to access the content. Our goal from the outset of this project was to reach members of the multi-disciplinary and rapidly growing hazards and disaster field, including students and early career researchers and practitioners. Accordingly, we worked to widely disseminate the training module. We wrote and shared an announcement through the Natural Hazards Center’s online news publication and social media accounts. We also shared brief announcements through various listservs, professional networks, and contacts across the social sciences, public health and medicine, engineering, and natural sciences disciplines. Additionally, we worked with the CDC TRAIN Learning Network to list the training module as a course on their website so that we could ensure its ongoing availability to public health practitioners. 

Within a month of its release, we also hosted a 30-min webinar where more than 400 researchers and practitioners signed up to view a demonstration of the module. The recorded and captioned video of the webinar was then shared on our project website (https://converge.colorado.edu/communications/webinar-series/) and disseminated again through various listservs and professional networks. In addition to these efforts, we continue to share the module regularly through presentations at academic and professional conferences, training sessions, and other forums. 

### 2.5. Evaluation and Analysis

To evaluate the training module, we assessed both the reach and effectiveness of the training. We determined program reach by examining whether we successfully accessed members of our target audience of a diverse hazards and disaster workforce, with a particular emphasis on students and those new to the field. At registration, trainees were asked to provide answers to a series of questions regarding their demographic characteristics and background (e.g., student status; researcher category; discipline; professional affiliation; racial and ethnic identity; gender identity; age; highest level of education; and geographical location). Provision of this information was voluntary, and trainees were provided with a written description informing them that no individual personal information would be shared with anyone outside of the CONVERGE research and evaluation team. Additionally, we sent a follow-up survey to all trainees to gather information on their disciplinary background and whether or not they had any previous advanced training or experience in disaster mental health. Descriptive statistics were generated for all trainee sociodemographic variables.

To evaluate training effectiveness, we examined whether trainees reported an increase in knowledge, skills, and attitude (KSA) related to disaster mental health before and after completing the module. KSA is a theoretically-informed framework used to evaluate competencies gained via educational trainings and in other educational or ork settings. Grounded in psychological research, learning outcomes can be classified as cognitive, skill based, and affective [40]. Cognitive outcomes relate to the quantity and types of knowledge gained. Skill-based outcomes concern the development of technical or motor skills. Affective outcomes focus on attitude and motivational outcomes that influence the choice of an action [40]. Together, these KSA outcomes have been used to evaluate multi-dimensional learning across different types of trainings, including those targeting the preparedness and response workforce. For example, the KSA framework has been used to assess readiness among disaster volunteers [41], as well as in CDC-funded Preparedness and Emergency Response Learning Centers for the evaluation of preparedness training and curricula for public health practitioners [42]. 

To measure KSA, all trainees (census sample) were required to answer the following assessment questions before and after completing the module:

On a scale from 1 (lowest) to 10 (highest):

1. Please rate your knowledge about disaster mental health.
2. Please rate your methodological skill set for conducting research on disaster mental health.
3. How important do you think building mental health knowledge and skills is to hazards and disaster research?

These questions were reviewed by subject matter experts at the CDC, as well as graduate students and research associates at the Natural Hazards Center before being disseminated. The questionnaire was integrated into the module so that all trainees were required to answer the three questions before starting the training. At the end of the module, before accessing the final quiz to receive a certificate of completion, all trainees were required to answer the same three questions as a follow-up assessment. Prior to answering these questions, trainees were informed that the responses to these questions would be used for evaluation purposes.

We conducted Wilcoxon Signed Rank tests to evaluate overall changes in perceived knowledge, skills, and attitudes between pre- and post-assessments for all participants. To explore how trainees’ sociodemographic characteristics and professional background influenced changes in KSA, we conducted Kruskal–Wallis tests for each of the KSA outcomes by student status; researcher category; racial and ethnic identity; geographical location; highest degree completed; gender identity; and affiliated organization. All statistical analyses were conducted in IBM SPSS 26.

## 3. Results

From its release on 8 October 2019, through 8 September 2020, 317 trainees completed the Disaster Mental Health Training Module. Descriptive statistics for the trainee sociodemographic characteristics and professional background are shown in Table 1. The majority of trainees identified as students (76.3%), White (65.9%), female (73.20%), and were affiliated with an academic institution (61.80%). A little over one-third of trainees reported their highest completed level of education as a bachelor’s degree (32.80%), followed by high school/GED (24.9%), and master’s degree (19.9%). While the vast majority of trainees indicated the United States (83.20%) as their country location, a limited number of trainees also resided in eleven other countries including Turkey, Canada, India, United Kingdom, Barbados, New Zealand, Australia, Bangladesh, Nepal, Saudi Arabia, and Syria. Roughly half of the trainees reported their age to be between 16 and 25 years (48.3%), with an average age of 26.45 years.

Users were also asked to indicate their level of involvement in the hazards or disaster field by self-identifying as either a core, periodic, situational, or emerging researcher or practitioner [16]. The “core” category includes those who strongly self-identify as a hazards/disaster researcher or practitioner, have engaged in hazards and disaster work for a sustained amount of time, and have a deep commitment to the field. The “emerging” category includes students and those who are new to the field, have limited experience, and are still learning about the disciplinary or interdisciplinary histories, theories, methods, and approaches in the hazards and disaster field. The “periodic” category includes those who are not primarily engaged in hazards and disaster research or practice but who focus on related topics from time to time throughout their professional career. Lastly, the “situational” category includes those not previously trained or involved in the hazards field, but who have had the opportunity to study new phenomena or processes based on a situational event. The majority of trainees for this module self-identified as emerging researchers or practitioners (65.9%).

Wilcoxon Signed Rank tests evaluating whether there were statistically significant differences in perceived knowledge, skills, and attitudes between pre- and post-assessments for all respondents are shown in Table 2. The results demonstrated a significant increase across all three of these measures, with median increases of 3.00 points for self-rated knowledge, 4.00 for self-rated skills, and 1.00 point in self-rated attitude. 

Kruskal–Wallis tests were performed to examine how participant sociodemographic characteristics and professional background influenced self-reported changes in knowledge, skills, and attitudes (Table 3). Overall, students reported a greater change in knowledge (mean rank = 176.08) compared to non-students (mean rank = 103.90), and this difference was statistically significant (H = 36.30, *p* < 0.001). Similarly, there was a statistically significant and greater change in knowledge (H = 6.97, *p* < 0.01) for respondents located in the United States (mean rank = 165.16) than for those located in other countries (mean rank = 129.65). Group differences in change in knowledge across researcher/practitioner category were also highly significant (H = 27.87, *p* < 0.001), with emerging researchers/practitioners reporting the greatest change (mean rank = 175.70) and core researchers reporting the lowest change (102.17). Significant differences across education (highest degree) were also found (H = 50.94, *p* < 0.001), as respondents with high school/GED education levels reported the greatest change (mean rank = 202.08), followed by bachelor’s degree (mean rank = 169.92), associate’s degree (mean rank = 158.17), master’s degree (mean rank = 113.44), and doctoral degree (mean rank = 108.55). We also found a marginally significant difference in knowledge change across gender identity (H = 6.44, *p* < 0.05), with respondents who responded with an identity other than male or female (which includes both non-conforming/non-binary and those who preferred not to answer) reporting the greatest change between pre- and post-assessments (mean rank = 182.34). Lastly, respondents affiliated with an academic institution reported the highest mean change in knowledge (mean rank = 135.61) while those affiliated with local, state, or federal governments reported the lowest mean change (mean rank = 83.17), and the group differences were statistically significant (H = 12.57, *p* < 0.01). Significant differences in knowledge change were not found across racial and ethnic categories.

In terms of changes in skill, students again reported a greater change (mean rank = 166.42) compared to non-students (mean rank = 136.07), and this difference was statistically significant (H = 6.84, *p* < 0.01). Likewise, there was a statistically significant change in skill (H = 11.04, *p* < 0.05) across researcher categories, with emerging researchers/practitioners reporting the greatest change in skill (mean rank = 168.90) and core researchers reporting the lowest change (mean rank = 121.84). Finally, group differences in change in skill across educational background were also significant (H = 16.28, *p* < 0.01), with respondents with a high school/GED education level reporting the greatest change (mean rank = 185.38) compared to the other degree categories. Group differences in skill change across affiliation, racial and ethnic group, gender, and geographical location were not significant.

None of the sociodemographic variables were significantly associated with changes in self-reported attitudes. 

In addition to the analyses above, we also generated descriptive statistics for the responses to the two follow-up questions we sent to trainees. While our response rate for this follow-up survey was too low (23%) to include these variables in the statistical analyses above, we found that 63.5% of those who responded did not have any advanced training or experience in disaster mental health prior to completing the module. The majority of participants came from the social sciences (51.4%), followed by public health (12.2%) and medicine/nursing (9.5%). Other disciplines represented included public administration, engineering, and the natural sciences.

## 4. Discussion

This article describes the development and dissemination of the CONVERGE Disaster Mental Health Training Module and evaluates changes in trainee knowledge, skills, and attitudes towards disaster mental health research after completing the module. Our evaluation of the module and its impact on the diverse user base allows us to assess progress in educating current and future members of the hazards and disaster workforce while ensuring that they are prepared to meet rising 21st-century environmental and social challenges [14,15]. 

In the eleven months since its release, 317 trainees completed the module. Our evaluation revealed that the majority of the trainees identified as female, White, students, and early career researchers/practitioners. The majority of people who completed the module were between the ages of 16 and 25 years. Most trainees live in the United States, although people from 11 additional countries also completed the module. Results from our follow-up survey also suggest that the trainees come from a range of disciplinary backgrounds, the majority of whom have no advanced training in disaster mental health. 

These findings suggest that we have been successful at reaching particular groups who have often been overlooked in hazards and disaster research and emergency management practice. Women, whose role has been limited in DRR [43] and whose capacities have often been minimized in disaster management [44,45], made up 73.2% of the trainees who completed our module. Young adults, who have also been understudied and less involved in DRR efforts, also made up the majority of our trainees. In fact, 76.3% of the trainees identified as students, and the average age of our trainees was 26.45 years. This represents an important response to recent calls to better engage young people in the hazards field and to acknowledge their energy, creativity, and capacity to contribute to DRR [46,47,48,49].

While disaster mental health research is often conducted in the realm of the social and behavioral sciences and public health and medicine, the trainees who completed our module came from a range of additional disciplines. This is an important finding, which offers early promise in terms of bridging the gap between disciplines traditionally engaged in risk reduction activities and those in the disaster mental health field [12]. Indeed, by training students and other emerging professionals across disciplines, this intervention can help advance a disaster mental health framework in the broader hazards and disaster field. Consider, for instance, early career engineers, emergency managers, and social scientists. Engineers, who often conduct post-disaster damage assessments, must be prepared to interact with survivors exposed to trauma [50]. Emergency managers are responsible for planning for and coordinating the disaster response and should be aware of available evidence-based resources to address population mental health needs. Social scientists study how social factors influence disaster outcomes and require the methodological skills to study disaster mental health. Training these audiences is necessary to advancing the field and ensuring mental health knowledge is better integrated into DRR. Moreover, because of the module’s focus on practical applications to reduce mental health distress among vulnerable populations, the module can help current and future members of the rapidly growing hazards and disaster workforce make connections between DRR and disaster mental health. 

Overall, we found that trainees reported significant increases in knowledge, skills, and attitudes regarding disaster mental health before and after completing the module. On average, trainees experienced the greatest change in methodological skills to conduct disaster mental health research. Given the widespread lack of appropriate, empirically based guidance for mental health service professionals to engage in DRR practices [12,51,52,53], this finding is encouraging as it suggests that the module can improve skills needed to advance research and practice. By providing trainees with the tools needed to study disaster mental health, the Disaster Mental Health Training Module can help to encourage researchers and practitioners to conduct research in this area and ultimately guide the development of disaster risk reduction practices and policies that center on disaster mental health.

Considering the increasing disciplinary, professional, and demographic diversity of hazards and disaster researchers [15,16,18], we were particularly interested in understanding how trainee sociodemographic characteristics and professional background influenced changes in knowledge, skills, and attitudes. Notably, we found student status, researcher category, and educational background to be significantly associated with changes in both knowledge and skills. Particularly, students, emerging researchers or practitioners, and those with lower levels of education (i.e., those who indicated their highest degree completed as high school/GED or a bachelor’s degree) experienced the greatest increases in knowledge and skills. In other words, those with little experience in the field and/or who are early in their career received the greatest benefit from the module. This finding is not entirely surprising given that those with minimal experience or less advanced education have the most potential to learn from this training. Still, it shows the growth that can occur even in the context of a relatively brief online training. Moreover, in line with recent calls for more sustained incorporation of mental health and psychosocial well-being into DRR practices [9,10], an important element of the education and training of the workforce must include a comprehensive understanding of disaster mental health. Our results, therefore, indicate that the Disaster Mental Health Training Module is adept at helping build this foundational knowledge and skills in the next generation of scholars and practitioners, as well as prepare them for the challenges they may face in the field.

Although the results of this evaluation are promising, there are limitations to our study that need to be addressed. First, the training module was released over a year ago and continues to be available for free online. As such, the results that we present in this article are preliminary, as more users complete the module each week. As the user base expands and diversifies, there is a chance that some of the patterns that we have shared in this article could change. Second, while analyzing the evaluation data, we realized that the initial registration form neglected to collect information on primary discipline and whether or not trainees had extensive experience or advanced training in disaster mental health. We felt this information was important for understanding our user base and thus sent a follow-up email and three additional reminders asking all trainees to provide answers to these questions. Only 74 of 317 trainees provided responses. We were therefore unable to include these variables in additional analyses, although we do infer patterns from the data we attained. We therefore acknowledge that the descriptive statistics presented for primary discipline and previous training in disaster mental health are not generalizable to our entire user base. Third, our evaluation design does not allow for assessing the longer-term career trajectories of the majority student population that we assessed for this evaluation project. We therefore have no way of knowing whether they will use the knowledge attained in their future careers or if they will apply the lessons learned toward DRR approaches. Even with these limitations in mind, we hope this module is of use to others and that it can help advance the conversation started by Gray and colleagues [12] regarding the importance of connecting DRR and disaster mental health research and practice. 

## 5. Conclusions

Despite a large body of literature examining the mental health consequences of disasters and large-scale emergencies, there is a need for more explicit incorporation of mental health and psychosocial well-being research into disaster risk reduction practices. Training and education programs, such as the CONVERGE Disaster Mental Health Training Module, can build important bridges between academic research, mental health and psychosocial service providers, and the disaster risk reduction and emergency management fields. By helping trainees build foundational knowledge of disaster mental health risk factors and outcomes, acquire the methodological skills necessary to conduct this research, and recognize the enduring challenges and questions that are central to the future of the field, our module provides actionable guidance for researchers and professionals to engage in DRR practices that aim to anticipate and reduce the mental health impacts of disasters and, ultimately, increase the resilience of people and communities. 

## Figures and Tables

**Table 1:** Sociodemographic Characteristics of CONVERGE Disaster Mental Health Training Module Trainees.

Variable	*N* (%)
**Student Status**	
Yes	242 (76.3%)
No	75 (23.7%)
**Race and Ethnicity**	
White	209 (65.9%)
Hispanic	21 (6.6%)
Asian	18 (5.7%)
Black	16 (5.0%)
Other ^1^	13 (4.2%)
More than 1 Race	11 (3.5%)
Prefer Not to Answer	39 (9.1%)
**Geographical Location by Country**	
United States	264 (83.2%)
Turkey	32 (10.1%)
Canada	5 (1.6%)
India	4 (1.3%)
United Kingdom	3 (0.9%)
Barbados	2 (0.6%)
New Zealand	2 (0.6%)
Australia	1 (0.3%)
Bangladesh	1 (0.3%)
Nepal	1 (0.3%)
Saudi Arabia	1 (0.3%)
Syria	1 (0.3%)
**Highest Degree Completed**	
High School/GED	79 (24.9%)
Associate’s	30 (9.5%)
Bachelor’s	104 (32.8%)
Master’s	63 (19.9%)
Doctorate	30 (9.5%)
Prefer Not to Answer	11 (3.5%)
**Gender Identity**	
Female	232 (73.2%)
Male	73 (23.0%)
Non-Binary/Non-Conforming	5 (1.6%)
Prefer Not to Answer	7 (2.2%)
**Affiliated Organization**	
Academic Institution	196 (61.8%)
Federal, State, or Local Government	35 (11.0%)
Other ^2^	86 (27.1)
**Age Range**	
16–25	153 (48.3%)
26–35	68 (21.5%)
36–45	26 (8.2%)
46–55	17 (5.4%)
56–65	10 (3.2%)
66+	8 (2.5%)
No Answer	35 (11%)
**Mean Age (Standard Deviation)**	26.45 years (12.19)
**Researcher Category**	
Core	47 (14.8%)
Emerging	209 (65.9%)
Periodic	33 (10.4%)
Situational	28 (8.8%)

^1^ “Other” consists of respondents who indicated their race or ethnicity as Arab, Indigenous, Native-Hawaiian, or Turkish through self-selecting pre-existing categories or writing in another identifier. ^2^ Due to small sample sizes, respondents who indicated they had an affiliation other than government or academic were condensed into “Other.” This included those from the private and non-profit sectors as well as retirees.

**Table 2:** Wilcoxon Signed Ranked Tests Assessing Differences in Perceived Knowledge, Skills, and Attitudes before and after Completing the CONVERGE Disaster Mental Health Training Module.

Outcome	Median Score Before	Median Score After	*p* Value
Knowledge	5.00	8.00	<0.001
Skills	3.00	7.00	<0.001
Attitudes	9.00	10.00	<0.001

**Table 3:** Kruskal–Wallis Tests for Change in Knowledge, Skills, and Attitudes across Participant Sociodemographic Characteristics.

**Student Status**	**Mean Rank Knowledge Change**	**Mean Rank Skills** **Change**	**Mean Rank Attitude** **Change**
Yes	176.08	166.42	162.18
No	103.90	135.07	148.74
**Kruskal–Wallis H**	36.30 ***	6.83 **	1.43
**Researcher Category**	**Mean Rank Knowledge Change**	**Mean Rank Skills** **Change**	**Mean Rank Attitude** **Change**
Core	102.17	121.84	153.70
Emerging	175.70	168.90	161.08
Periodic	135.05	160.94	156.15
Situational	158.00	145.18	155.75
**Kruskal–Wallis H**	27.87 ***	11.04 *	0.39
**Race and Ethnicity**	**Mean Rank Knowledge Change**	**Mean Rank Skills** **Change**	**Mean Rank Attitude** **Change**
White	158.48	159.04	159.60
All Other ^1^	160.00	158.92	157.83
**Kruskal–Wallis H**	0.02	0.00	0.03
**Geographical Location**	**Mean Rank Knowledge Change**	**Mean Rank Skills** **Change**	**Mean Rank Attitude** **Change**
U.S.	165.16	163.11	160.89
All Other ^2^	129.65	139.43	149.99
**Kruskal–Wallis H**	6.97 **	3.10	0.75
**Highest Degree**	**Mean Rank Knowledge Change**	**Mean Rank Skills** **Change**	**Mean Rank Attitude** **Change**
High School/GED	202.08	185.38	172.93
Associate’s	158.17	155.50	161.47
Bachelor’s	169.92	166.34	160.45
Master’s	113.44	134.73	138.10
Doctorate	108.55	129.75	146.73
Prefer Not to Answer	147.18	128.50	191.68
**Kruskal–Wallis H**	44.75 ***	16.28 **	8.25
**Gender Identity**	**Mean Rank Knowledge Change**	**Mean Rank Skills** **Change**	**Mean Rank Attitude** **Change**
Female	164.98	164.91	158.38
Male	136.16	142.20	161.26
All Other ^3^	182.38	147.00	157.25
**Kruskal–Wallis H**	6.44 *	3.70	0.07
**Affiliated Organization**	**Mean Rank Knowledge Change**	**Mean Rank Skills** **Change**	**Mean Rank Attitude** **Change**
Academic	168.94	158.87	161.20
Government	114.39	139.00	136.86
All Other ^4^	154.50	167.44	163.00
**Kruskal–Wallis H**	11.05 **	2.45	2.70

Notes: ^1^ Due to small sample sizes for specific race and ethnic categories, race was recoded into a dichotomous variable to indicate whether or not the respondent indicated their race as White. ^2^ Due to small sample sizes, respondents who indicated their location outside of the U.S. were recoded into “Other” category. ^3^ Due to small sample sizes, respondents who indicated their gender identity as non-binary/non-conforming or prefer not to answer were recoded into “Other” category. ^4^ Due to small sample sizes, respondents who indicated they had an affiliation other than government or academic were condensed into the “Other” category. * *p* < 0.05; ** *p* < 0.01; *** *p* < 0.001.

## Data Availability

The de-identified data presented in this study are available on request from the corresponding author. The data are not publicly available due to privacy concerns and the dynamic nature of the dataset.

## References

[1] Beaglehole B., Mulder R.T., Frampton C.M., Boden J.M., Newton-Howes G., Bell C.J. (2018). Psychological distress and psychiatric disorder after natural disasters: Systematic review and meta-analysis. Br. J. Psychiatry.

[2] Centers for Disease Control and Prevention Learn about Mental Health. https://www.cdc.gov/mentalhealth/learn/index.htm.

[3] Goldmann E., Galea S. (2014). Mental Health Consequences of Disasters. Annu. Rev. Public Health.

[4] Lai B.S., Esnard A.M., Lowe S.R., Peek L. (2016). Schools and disasters: Safety and mental health assessment and interventions for children. Curr. Psychiatry Rep..

[5] Norris F.H., Friedman M.J., Watson P.J., Byrne C.M., Diaz E., Kaniasty K. (2002). 60,000 disaster victims speak: Part I. An empirical review of the empirical literature, 1981–2001. Psychiatry: Interpers. Biol. Process..

[6] Fothergill A., Peek L.A. (2004). Poverty and disasters in the United States: A review of recent sociological findings. Nat. Hazards.

[7] La Greca A.M., Lai B.S., Joormann J., Auslander B.B., Short M. (2013). Children’s risk and resilience following a natural disaster: Genetic vulnerability, posttraumatic stress, and depression. J. Affect. Disord..

[8] Pollack A.A., Weiss B., Trung L.T. (2016). Mental health, life functioning and risk factors among people exposed to frequent natural disasters and chronic poverty in Vietnam. Bjpsych Open.

[9] United Nations International Strategy for Disaster Reduction (2015). Sendai Framework for Disaster Risk Reduction 2015–2030.

[10] World Health Organization (2019). Health Emergency and Disaster Risk Management Framework.

[11] Mileti D. (1999). Disasters by Design: A Reassessment of Natural Hazards in the United States.

[12] Gray B., Hanna F., Reifels L. (2020). The integration of mental health and psychosocial support and disaster risk reduction: A mapping and review. Int. J. Environ. Res. Public Health.

[13] Norris F.H., Friedman M.J., Watson P.J. (2002). 60,000 disaster victims speak: Part II. Summary and implications of the disaster mental health research. Psychiatry: Interpers. Biol. Process..

[14] National Research Council (2006). Facing Hazards and Disasters: Understanding Human Dimensions.

[15] Peek L., Tobin J., Adams R., Wu H., Mathews M. (2020). A framework for convergence research in the hazards and disaster field: The Natural Hazards Engineering Research Infrastructure CONVERGE Facility. Front. Built Environ..

[16] Peek L., Champeau H., Austin J., Mathews M., Wu H. (2020). What methods do social scientists use to study disasters? An analysis of the Social Science Extreme Events Research (SSEER) network. Am. Behav. Sci..

[17] Madrigano J., Chandra A., Costigan T., Acosta J.D. (2017). Beyond disaster preparedness: Building a resilience-oriented workforce for the future. Int. J. Environ. Res Public Health.

[18] Moseley L. (2004). Educational needs for disaster management. Aust. J. Emerg. Manag..

[19] Neal D.M. (2000). Developing degree programs in disaster management: Some reflections and observations. Int. J. Mass Emergencies Disasters.

[20] Peek L. (2006). Transforming the field of disaster research through the next generation. Int. J. Mass Emergencies Disasters.

[21] Louis-Charles H., Dixon B. (2015). A blueprint for change: An emerging initiative paves the way for increased diversity in hazards mitigation. Nat. Hazards Obs..

[22] National Academies of Sciences, Engineering, and Medicine (2020). Evidence-Based Practice for Public Health Emergency Preparedness and Response.

[23] Saraceno B., van Ommeren M., Batniji R., Cohen A., Gureje O., Mahoney J., Sridhar D., Underhill C. (2007). Barriers to improvement of mental health services in low-income and middle-income countries. Lancet.

[24] Adams R.M., Evans C., Peek L. Social vulnerability and disasters. CONVERGE Social Vulnerability and Disasters Training Module. https://converge.colorado.edu/resources/training-modules.

[25] Young B.H., Ruzek J.L., Pivar I. (2001). Mental Health Aspects of Disaster and Community Violence: A Review of Training Materials.

[26] Young B.H., Ruzek J.I., Wong M., Salzer M.S., Naturale A.J., Ritchie E.P., Watson P.J., Friedman M.J. (2005). Disaster mental health training. Interventions Following Mass Violence and Disasters: Strategies for Mental Health Practice.

[27] Vernberg E.M., Steinberg A.M., Jacobs A.K., Brymer M.J., Watson P.J., Osofsky J.D., Layne C.M., Pynoos R.S., Ruzek J.I. (2008). Innovations in disaster mental health: Psychological first aid. Prof. Psychol. Res. Pract..

[28] American Red Cross Disaster Training. https://www.redcross.org/take-a-class/disaster-training.

[29] McFarlane A.C., Norris F.H., Norris F.H., Galea S., Friedman M.J., Watson P.J. (2006). Definitions and concepts in disaster research. Methods for Disaster Mental Health Research.

[30] Wang C.W., Chan C.L., Ho R.T. (2013). Prevalence and trajectory of psychopathology among child and adolescent survivors of disasters: A systematic review of epidemiological studies across 1987–2011. Soc. Psychiatry Psychiatr. Epidemiol..

[31] Abramson D.M., Stehling-Ariza T., Garfield R.M., Redlener I.E. (2018). Prevalence and predictors of mental health distress post-Katrina: Findings from the Gulf Coast Child and Family Health Study. Disaster Med. Public Prep..

[32] North C.S., Pfefferbaum B. (2002). Research on the mental health effects of terrorism. JAMA.

[33] Paxson C., Fussell E., Rhodes J., Waters M. (2012). Five years later: Recovery from post traumatic stress and psychological distress among low-income mothers affected by Hurricane Katrina. Soc. Sci. Med..

[34] Picou J.S., Nicholls K. (2019). Caught in the Path of Katrina: A Survey of the Hurricane’s Human Effects.

[35] Fothergill A., Peek L. (2015). Children of Katrina.

[36] Palinkas L.A., Norris F.H., Galea S., Friedman M.J., Watson P.J. (2006). Qualitative approaches to studying the effects of disasters. Methods for Disaster Mental Health Research.

[37] McKinzie A.E. (2018). In their own words: Disaster and emotion, suffering, and mental health. Int. J. Qual. Stud. Health Well-Being.

[38] Centers for Disease Control and Prevention Helping Children Cope During and after a Disaster. https://www.cdc.gov/childrenindisasters/pdf/children-coping-factsheet-508.pdf.

[39] Watson P.J., Brymer M.J., Bonanno G.A. (2011). Postdisaster psychological intervention since 9/11. Am. Psychol..

[40] Kraiger K., Ford J.K., Salas E. (1993). Application of cognitive, skill-based, and affective theories of learning outcomes to new methods of training evaluation. J. Appl. Psychol..

[41] Meyer M.A., Peek L., Unnithan N.P., Coşkun R., Tobin-Gurley J., Hoffer K.H. (2016). Planning for diversity: Evaluation of a volunteer disaster response program. J. Cult. Divers..

[42] Centers for Disease Control and Prevention (2018). Knowledge, Skills, and Attitudes (KSAs) for the Public Health Preparedness and Response Core Competency Model.

[43] Hemachandra K., Amaratunga D., Haigh R. (2018). Role of women in disaster risk governance. Procedia Eng..

[44] Enarson E., Fothergill A., Peek L., Rodriguez H., Donner W., Trainor J.E. (2018). Gender and disasters. Handbook of Disaster Research.

[45] Sohrabizadeh S. (2016). The neglect of women’s capacities in disaster management systems in Iran: A qualitative study. Indian J. Gend. Stud..

[46] Mort M., Rodriguez-Giralt I., Delicado A. (2021). Children and Young People’s Participation in Disaster Risk Reduction.

[47] Peek L. (2008). Children and disasters: Understanding vulnerability, developing capacities, and promoting resilience. Child. Youth Environ..

[48] Peek L., Abramson D., Cox R., Fothergill A., Tobin J., Rodriguez H., Donner W., Trainor J.E. (2018). Children and disasters. Handbook of Disaster Research.

[49] Wisner B. (2006). Let Our Children Teach Us! A Review of the Role of Education and Knowledge in Disaster Risk Reduction. A Report by the ISDR System Thematic Cluster/Platform on Knowledge and Education.

[50] Bermudez Tapia B., Fehr T., Niles S., Evans C.M., Adams R.M., Peek L. CONVERGE Conducting Emotionally Challenging Research Training Module. https://converge.colorado.edu/resources/training-modules.

[51] Eisenman D., Weine S., Green B., Jong J.D., Rayburn N., Ventevogel P., Keller A., Agani F. (2006). The ISTSS/Rand guidelines on mental health training of primary healthcare providers for trauma-exposed populations in conflict-affected countries. J. Traumat. Stress.

[52] Langan J.C., Lavin R., Wolgast K.A., Veenema T.G. (2017). Education for developing and sustaining a health care workforce for disaster readiness. Nurs. Adm. Q..

[53] Yutrzenka B.A., Naifeh J.A. (2008). Traumatic stress, disaster psychology, and graduate education: Reflections on the special section and recommendations for professional psychology training. Train. Educ. Prof. Psychol..

